# Natural products for the treatment and management of diabetes mellitus in Zimbabwe-a review

**DOI:** 10.3389/fphar.2022.980819

**Published:** 2022-08-24

**Authors:** Remigio Usai, Stephen Majoni, Freeborn Rwere

**Affiliations:** ^1^ Department of Chemistry, Marquette University, Milwaukee, WI, United States; ^2^ Department of Chemical and Forensic Sciences, Botswana International University of Science and Technology, Palapye, Botswana; ^3^ Department Anesthesiology, Perioperative, and Pain Medicine, School of Medicine, Stanford University, Stanford, CA, United States; ^4^ Department of Chemistry, School of Natural Sciences and Mathematics, Chinhoyi University of Technology, Chinhoyi, Zimbabwe

**Keywords:** phytochemicals, diabetes mellitus, hyperglycemia, antidiabetic, streptozotocin

## Abstract

Use of medicinal plants and herbs in the treatment and management of diseases, including diabetes mellitus and its complications remains an integral part of African tradition. In Zimbabwe, nearly one million people are living with diabetes mellitus. The prevalence of diabetes mellitus in Zimbabwe is increasing every year due to lifestyle changes, and has accelerated the use of traditional medicines for its treatment and management in urban areas. In addition, the high cost of modern medicine has led many people in rural parts of Zimbabwe to rely on herbal plant medicine for the treatment of diabetes mellitus and its complications. This review highlights a number of studies carried out to evaluate the antidiabetic properties of indigenous plants found in Zimbabwe with the goal of treating diabetes mellitus. Further, we discuss the mechanism of action of various plant extracts in the treatment and management of diabetes mellitus. Together, this review article can open pathways leading to discovery of new plant derived medicines and regularization of use of crude plant remedies to treat diabetes mellitus by the Zimbabwean government and others across Africa.

## Introduction

Diabetes mellitus or diabetes is a chronic disease in which blood glucose, also referred to as blood sugar, becomes too high (Saeedi et al., 2019; Mohammed and Tajuddeen, 2022; Sun et al., 2022). Initially considered a disease of the Western world, diabetes mellitus is now a global pandemic that affects approximately 536.6 million people worldwide, and is predicted to rise to 643 million people by 2030 and 783.2 million people by 2045 (Saeedi et al., 2019; Lin et al., 2020; Sun et al., 2022). In 2021 the International Diabetes Federation (IDF) estimated that Africa had a diabetes mellitus prevalence of 23.6 million people (4.5%) and projected an increase of up to 5.2% (54.9 million people) in 2045 (Sun et al., 2022). Diabetes mellitus is characterized by hyperglycemia resulting from defects in insulin secretion, insulin action, or both (Tran et al., 2020; Mohammed and Tajuddeen, 2022). Hyperglycemia caused by a deficiency in insulin production by the β-cells of the pancreas is known as Type 1 diabetes mellitus (T1DM) and due to insufficiency of insulin production in the face of insulin resistance or β-cells dysfunction is Type 2 diabetes mellitus (T2DM) (Care, 2013; Tran et al., 2020). High blood glucose can lead to pathophysiologies including, heart disease, stroke, kidney disease, eye problems, dental disease, nerve damage and feet problems (Policardo et al., 2015; Bragg et al., 2017; Yang et al., 2019). Insulin is the most common type of medication prescribed in type 1 diabetes mellitus treatment and can also be used in some type 2 diabetes mellitus cases. Nearly 65 drugs are clinically approved to lower blood sugar in order to limit the occurrence of Type 2 diabetes-related pathophysiologies. However, anti-diabetic drugs have also shown a vast array of side effects including diarrhea, nausea, vomiting, heartburn and lactic acidosis (Ashraf et al., 2022).

Approximately four billion people in developing countries depend on herbal traditional medicine for the treatment of metabolic diseases such as diabetes mellitus because of the presence of a wide range of bioactive phytochemical compounds in plants (Ekor, 2014; Choudhury et al., 2018). In addition, plant-derived drugs such as metformin from *Galega officinalis* plant are commercially marketed for diabetes mellitus treatment (Bailey, 2017). Plant-based traditional medicines are considered to be cheap and readily available to the majority of the rural population in Africa (Mugumbate et al., 2018). In Zimbabwe, it was estimated that ∼850,000 people (about 5.7% of the population) are living with diabetes mellitus (Mutowo et al., 2015) and the average cost of conventional treatment of diabetes mellitus was a little over $1,300 United States Dollars (USD) (Mutowo et al., 2016). A study at a major hospital in Harare, the capital city of Zimbabwe showed that only 41.8% (76 of 182 diabetic patients) had good glycemic control using conventional medication (Chirombe et al., 2018). This has resulted in the majority of patients using medicinal plant-based treatment in combination with their conventional treatment. In addition, for over two decades, Zimbabwe has experienced an economic downturn that has led many people to rely on plant-derived medicines for treatment of many ailments, including diabetes mellitus due to high cost of modern medicine, and lack of foreign currency to procure drugs (Mutowo et al., 2016). Hence, much attention is needed to understand medicinal plants and their potential bioactive phytochemicals. Furthermore, there is an increased concern for safety and drug resistance from continued use of modern drugs. Despite their widespread use, natural plant products have some drawbacks including the presence of potential carcinogenic agents in some of these plants and complexity of the intrinsic metabolites making them unsuitable for therapeutic applications (Fennell et al., 2004). Also, traditional healers and herbalists do not possess adequate knowledge to understand the active components of the plant extracts and their mechanism of action. As such, it is important to understand and document the role of plant-derived bioactive phytochemicals in regulating blood glucose levels and diabetes mellitus treatment in Zimbabwe.

For many years, traditional medicines from plant extracts have proven to be clinically effective in the treatment of chronic ailments (Cock et al., 2019; Cock and Van Vuuren, 2020; Mohammed and Tajuddeen, 2022). In several cultures across Africa, there is widespread traditional use of decoctions prepared from medicinal plants in the treatment of diabetes mellitus (Mohammed and Tajuddeen, 2022). Use of decoctions in the treatment of complex diseases such as diabetes mellitus is important because plants contain many bioactive phytochemical compounds with various beneficial biological effects, thus potentially creating an effective and affordable multi-targeted treatment strategy. Diabetes mellitus has been linked to oxidative stress which arises from the excessive production of free radicals in the mitochondrial electron transport chain (Giacco and Brownlee, 2010; Asmat et al., 2016). Phytochemicals, including polyphenols and flavonoids have antioxidant properties and can scavenge free radicals and reduce oxidative stress, leading to treatment of diabetes mellitus (Johansen et al., 2005; Lv et al., 2021). They exert anti-hyperglycemic effects by binding to glucose transporters and competitively inhibiting the digestive enzymes (α-amylase and α-glucosidase). Other secondary plant metabolites such as terpenes, alkaloids, and saponins may enhance insulin secretion and regulate glucose uptake and glucose utilization. Bioactive phytochemicals can also exert antidiabetic effects by improving the performance of pancreatic tissue, often done by increasing insulin secretions or reducing the intestinal absorption of glucose by inhibiting key enzymes involved in glucose production (Kooti et al., 2016). Therefore, it is important to document plants with bioactive phytochemicals with antioxidant properties and are capable of inhibiting key enzymes (including α-amylase and α-glucosidase) to treat diabetes mellitus in traditional systems.

In this review, we discuss important medicinal plants found in Zimbabwe with the goal of treating type 2 diabetes mellitus and its management in rural communities. In addition, we will discuss how different plant extracts regulates blood glucose and their mechanism of action. Selection of plants for this study was based on indigenous knowledge related to plants used in Zimbabwe for the treatment of diabetes mellitus and its complications. However, there is limited research in Zimbabwean literature to justify the pharmacological use of some of these indigenous plants. Here, we summarize research findings for plants indigenous to Zimbabwe but investigated in Zimbabwe or other parts of the world. The summarized research was obtained from exhaustive search of plants on international databases, including Wiley library, Google Scholar, PubMed, SciFinder, Science Direct, Scopus and Springer Link using plant names and key words such as antidiabetic and hyperglycemia. Taken together with the present findings, research focusing on medicinal plants could ultimately lead to discovery of novel antidiabetic compounds to treat high blood sugar for low-income countries, and ultimately improve the healthcare of marginalized rural people with diabetic complications.

## Medicinal plants in Zimbabwe with antidiabetic properties

**FIGURE 1:**
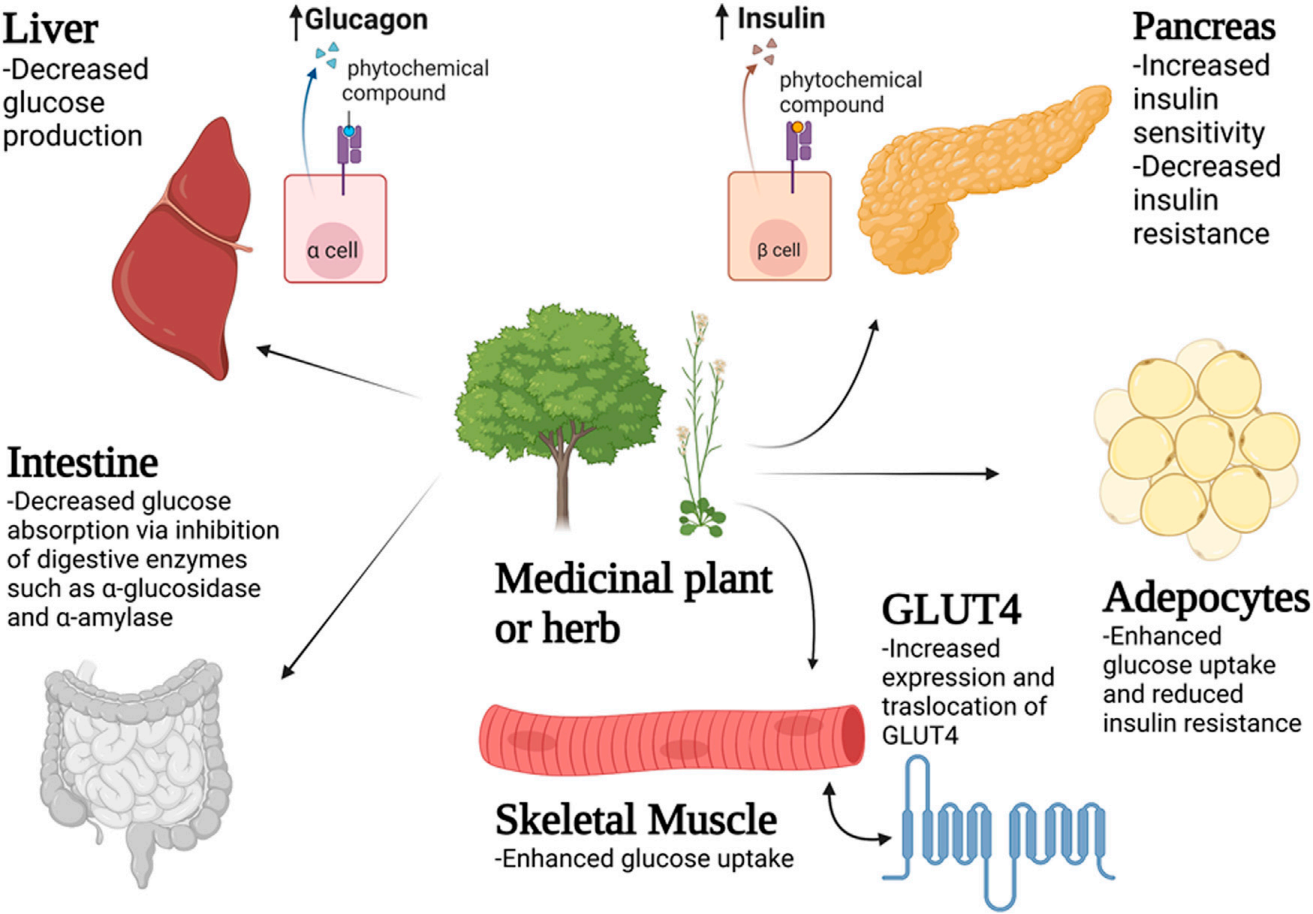
The mechanism of decreasing blood glucose levels by medicinal plants and herbs on different tissues and organs such as the liver, intestine, pancreas, skeletal muscle and adipose tissues.

Over the years, there has been an increase in the emergence of multidrug, extreme and total drug resistant pathogens. Resistance to current drugs, insufficient/incompatible therapies and negative side effects associated with some of the currently used drugs have severely affected the management of diabetes mellitus in many parts of Africa (Care, 2013). Various categories of antidiabetic drugs available on the market for treatment and management of diabetes mellitus include insulin analogues, sulphonylureas, biguanides, dipeptidyl peptidase-4 inhibitors, thiazolidinediones and ⍺-glucosidase inhibitors. However, the cost of these modern drugs has been on an upward trend worldwide. In addition, conventional diabetic therapies using modern medicines have resulted in increased side effects for many patients. As a result of these issues, there is a growing interest in the use of herbal remedies for the treatment and management of diabetes mellitus as they are perceived to be cheaper and with fewer side effects when compared to modern medicines. Table 1 summarizes the medicinal plants in Zimbabwe with antidiabetic properties used in this review, while Figure 1 presents the mode of action exerted by these plants in decreasing blood glucose levels.

**TABLE 1:** Medicinal plants in Zimbabwe with antidiabetic properties and their pharmacological outcomes.

Botanical name	Family	Image	Medicinal part/s used	Type of extract	Animal model	Pharmacological outcome/s
*Cassia abbreviata* Oliv*.*	Caesalpiniaceae	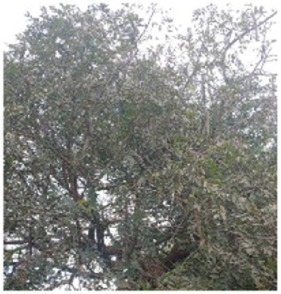	Stem bark	Aqueous, ethanol, acetone	Diabetic rats	1. Doses of *Cassia abbreviate* Oliv. normalized glucose level, and helped to maintain normal body weights in T2DM (Bati et al., 2017)
						2. The aqueous extracts enhanced glucose uptake and induced a two-fold increase in glucose transporter 4 (GLUT3) translocation in C2C12 mouse skeletal muscle cells (Kamga-Simo et al., 2021).
*Artemisia afra* Jacq. ex Willd	Asteraceae	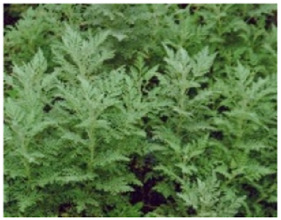	Leaf, Herbal tea	Aqueous, methanolic	Rats, Diabetic mice	1.Doses of *Artemisia afra* Jacq. ex Willd*.* extracts increased body weight, and decreased blood glucose level (Sunmonu and Afolayan, 2013).
						2. Blood glucose level in alloxan induced diabetic mice was decreased by 24.0% and 56.9%, in groups that received aqueous extracts. Methanolic extracts lowered glucose level by 49.8% (Issa and Bule, 2015).
						3. The blood glucose level was significantly reduced and insulin levels increased in diabetic rats fed with 100 mg/kg body weight aqueous extracts (Afolayan and Sunmonu, 2011).
						4. Aqueous extracts increased the levels of glutathione reductase, glutathione peroxidase, superoxide dismutase and glutathione in the liver and kidney of diabetic rats to normal levels and reduced the levels of lipid-peroxidation products (Afolayan and Sunmonu, 2013).
*Moringa oleifera* Lam.	Moringaceae	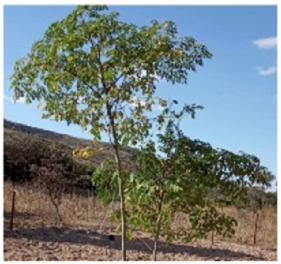	Stem bark, dried fruit powder, leaf, pods, seed powder	Ethanolic, methanol, aqueous	Alloxan Albino rats, STZ-induced diabetic rats, humans	1.The blood and urine glucose levels were significantly reduced by a single dose ethanolic extract (Kar et al., 2003)
						2. The pancreatic islets were rejuvenated after treatment of streptozotocin induced diabetic rats with methanol extracts of *Moringa oleifera* Lam. pods (Gupta et al., 2012; Al-Malki and El Rabey, 2015).
						3. Aqueous extracts of *Moringa oleifera* Lam. inhibited α-amylase and α-glucosidase with IC_50_ values of 52.5 and 33.4 mg/ml (Khan et al., 2017).
						4. *Moringa oleifera* Lam. powdered extracts and formulated cookies significantly reduced postprandial glucose in diabetic patients (Ahmad et al., 2018; Sissoko et al., 2020).
*Aloe vera* (L.) Burm.f	Asphodelaceae	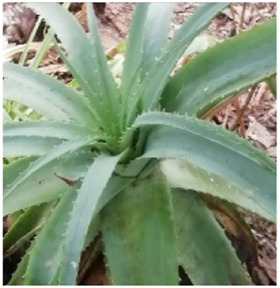	Gel, leaf	Methanol	STZ-induced diabetic rats, obesogenic WNIN/GR-Ob rats, Wistar rats, humans	1. A significant decrease in blood glucose level was observed with a dosage of 500 mg/kg body weight *Aloe vera* leaf pulp extract (Okyar et al., 2001).
						2. Blood glucose and insulin levels were restored in diabetic rats. The pancreatic islets of diabetic rats were improved (Najafian et al., 2019).
						3. The carbohydrate fraction of *Aloe vera* extract rejuvenated the pancreatic β-cells and improved insulin production. In addition, the fraction lowered fasting plasma glucose, glucagon and glucose-6-phosphatase levels in diabetic rats (Govindarajan et al., 2021).
						4. The methanolic extract significantly decreased the formation of advanced glycation end products (AGEs) and reduced the activities of *α*-amylase and *α*-glucosidase. The extract also increased the content of thiol groups (Muñiz-Ramirez et al., 2020).
						5. *Aloe vera* (L.) Burm.f extracts significantly reduced fasting blood glucose in diabetic and pre-diabetic patients (Devaraj et al., 2013; Alinejad-Mofrad et al., 2015).
*Psidium guajava* L.	Myrtaceae	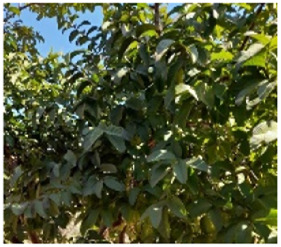	leaf	Aqueous, ethanol	Rats	1. Significant decrease in blood sugar levels was observed in diabetic rats treated with extract compared to control group (Shen et al., 2008; Musdja et al., 2017). Long term administration resulted in increased plasma insulin level and glucose utilization in diabetic rats (Shen et al., 2008).
						2 Aqueous extracts significantly lowered fasting plasma glucose levels and improved glucose tolerance and insulin sensitivity of diabetic mice. The extracts also altered the composition of the gut microbiota and increased the enrichment of probiotics (Chu et al., 2022).
						3.The fasting blood glucose and hemoglobin A1c (HbA1c) of the diabetic rats were decreased (Khan et al., 2013; Li et al., 2021).
						4. Leaf and bark extracts significantly inhibited α-glucosidase with IC_50_ values of 1.0 ± 0.3 and 0.5 ± 0.01 μg/ml, and α-amylase with IC_50_ values of 10.6 ± 0.4 μg/ml (Beidokhti et al., 2020).
						5. Bread fortified with *Psidium guajava* L. leaves attenuated diabetic and acute renal failure symptoms in diabetic rats (El-khalik, 2017).
*Persia Americana* Mill.	Lauraceae	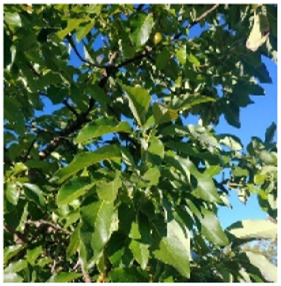	Leaf, seeds, fruit	Hydroethanolic, hydroalcoholic, aqueous	Streptozotocin (STZ)-induced diabetic Rats	1. Blood glucose levels and metabolic rates of animals were significantly improved. Activation of protein kinase B (PKB) was observed in the liver and skeletal muscle of treated rats compared with untreated rats. Possibly act to regulate glucose uptake in liver by PKB/Akt activation (Lima et al., 2012).
						2. The seed extracts promoted the activation of the PI3K/AkT pathway and inhibited β-cell death in diabetic rats (Ojo et al., 2022).
*Lippia javanica* (Burm.f.) Spreng*.*	Verbenaceae	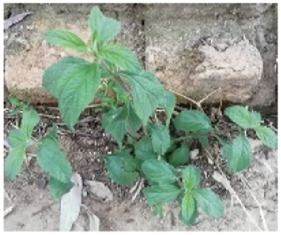	Herbal tea, leaf	Aqueous	Alloxan-induced diabetic mice	1. Aqueous extracts significantly lowered blood glucose levels in alloxan-induced diabetic mice (Arika et al., 2015).
*Parinari curatellifolia* Planch. ex Benth.	Chrysobalanaceae	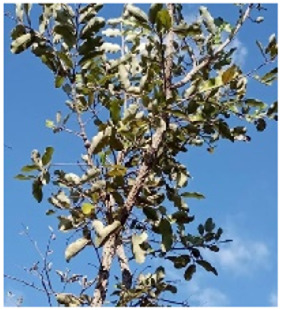	Seeds, stem bark, peel flour	Ethanolic	Alloxan-induced diabetic rats, *Drosophila melanogaster* flies	1. Plasma glucose and low-density lipoprotein (LPL)-cholesterol levels were significantly reduced in diabetic rats treated with ethanolic extracts relative to control group. Significant increase in high-density lipoprotein (HDL)-cholesterol was also observed in the treated group compared to control group (Ogbonnia et al., 2008, 2011).
						2. The ethanolic extract significantly reduced blood glucose, total thiol and nitric oxide levels of diabetic-induced *Drosophila melanogaster* flies. The extracts also increased glutathione-S-transferase and catalase activities of the diabetic treated flies (Omale et al., 2020).
						3. The *Parinari curatellifolia* Planch. ex Benth peel flour formulated biscuits have improved total phenolic and flavonoid content as well as antioxidant activity (Ramashia et al., 2021).
*Mangifera indica* L.	Anacardiaceae	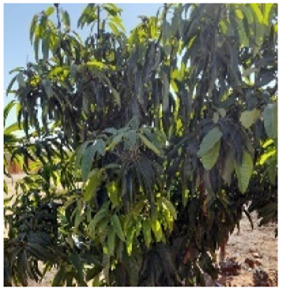	Leaf, fruit peel	aqueous	STZ-induced diabetic rats, Alloxan-induced diabetic mice	1. *Mangifera indica* L. supplemented diet reduced lipid peroxidation products in the cerebellum and cortex of diabetic rats (Cázares-Camacho et al., 2021).
						2. Significant decrease in glucose and leptin levels coupled with elevation in insulin levels and C-peptide were observed in diabetic rats (El-Sheikh, 2012).
						3. Remarkable decrease in postprandial blood glucose level was observed in diabetic mice. In addition, glucose tolerance and body weight and lipid profiles improved in diabetic treated mice. The extracts also decreased the damage to β-cells (Saleem et al., 2019).
						4. The extracts significantly inhibited α-glucosidase and α-amylase (Gondi and Prasada Rao, 2015; Ojo et al., 2018).
*Momordica charantia* L.	Cucurbitaceae	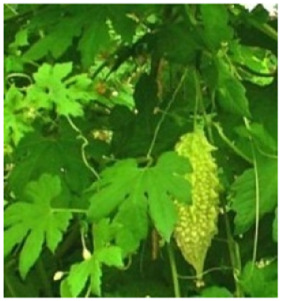	Fruit juice, skin, flesh, whole fruit	Aqueous	STZ-induced diabetic rats, Sprague Dawley rats, humans	1. Increased levels of serum insulin, HDL-cholesterol, total antioxidant capacity levels, β-cell function, and pancreatic reduced glutathione (GSH) content was observed with fruit juice administration (Mahmoud et al., 2017).
						2. The fruit juice reduced glycated hemoglobin A1c, blood glucose, body weight, BMI, fat percentage, and waist circumference in humans. The fruit juice also caused an increment of insulin area under curve (AUC), first phase and total insulin secretion (Cortez-Navarrete et al., 2018).
						3. A whole fruit juice resulted in 31.6% lowering of blood glucose level and 27.4% increase in insulin level in hyperglycemic rats (Mahwish et al., 2021)
						4. *Momordica charantia* L. fruit juice significantly reduced fasting blood glucose, body weight, HbA1c and blood serum glucose, and increase in insulin secretion of diabetic and prediabetic subjects (Cortez-Navarrete et al., 2018; Krawinkel et al., 2018; Majeed et al., 2021).
*Annona Stenophylla* Engl. and Diels	Annonaceae	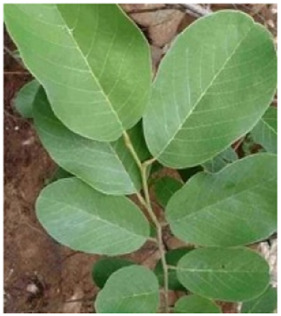	Roots, root bark	Ethanolic, Aqueous	Alloxan induced diabetic rats	1. Dose dependent decrease in plasma glucose levels of alloxan induced diabetic rats (Verengai et al., 2017) and increase in glucose uptake was observed in C2C12 myocytes treated with ethanolic extracts (Taderera et al., 2019).
						2. *Annona Stenophylla* Engl. and Diels extracts resulted in an increase in transcription of GLUT4 mRNA when compared with untreated control (Taderera et al., 2019).
						3. The extracts significantly inhibited α-glucosidase and α-amylase (Taderera et al., 2016).

### *Cassia abbreviata* oliv.

*Cassia abbreviata* Oliv. From the *Fabaceae* family is widely distributed across many parts of Southern Africa, including Zimbabwe. Although *Cassia abbreviata* Oliv. has been used in African traditional medicine for the treatment of diabetes mellitus, there are few pharmacological findings to justify its use as an antidiabetic medicinal plant. Here we summarize some of the recent studies that show its potential to act as an antidiabetic medicinal plant. (Kamga-Simo et al., 2021) assessed the potential of *Cassia abbreviata* Oliv. aqueous extracts to function as an antidiabetic agent on skeletal muscle cells and showed that the aqueous concoction enhanced glucose uptake and induced a two-fold increase in glucose transporter 4 (GLUT4) translocation in C2C12 mouse skeletal muscle cells. Real-time (RT) qPCR showed an increased expression of GLUT4, IRS1 (insulin receptor substrate 1) and PI3K (phosphoinositide 3-kinase) in cells treated with bark extract (Kamga-Simo et al., 2021). A study by (Shai et al., 2010) showed that acetone extracts of *Cassia abbreviata* Oliv. resulted in an 88% inhibition of yeast α-glucosidase activity at the study’s highest concentration with an IC_50_ of 0.6 mg/ml. Enzyme kinetic studies suggested non-competitive inhibition by the active components in the extracts. *Cassia abbreviata* Oliv. extracts also showed pronounced antioxidant activity (Shai et al., 2010).

(Bati et al., 2017) evaluated the antidiabetic effects of ethanolic bark extract of *Cassia abbreviata* Oliv. on diabetic albino rats and showed that two doses of the ethanolic bark extract normalized blood glucose levels and helped to maintain normal body weights of the rats. Additionally, it was observed that the ethanolic doses increased hexokinase and decreased glucose-6-phosphate activities in the liver and kidneys. The extracts also inhibited the activity of α-glucosidase and enhanced glucose uptake (Bati et al., 2017). Taken together, these studies indicate that bioactive phytochemical compounds and other secondary plant metabolites in *Cassia abbreviata* Oliv. crude extracts can be used in the lowering and management of diabetes mellitus with maximal effects and minimal side effects.

### Artemisia afra jacq. ex willd

*Artemisia afra Jacq.* ex Willd. or African wormwood is one of the most widely used medicinal plants in many parts of southern Africa because of its acclaimed healing properties against many ailments including diabetes mellitus (Dabe and Kefale, 2017). Concoctions of *Artemisia afra* Jacq. ex Willd. have also been used to treat the severe acute respiratory syndrome coronavirus 2 (SARS-CoV-2) or Covid-19 disease (Orege et al., 2021). For over a decade, studies have been carried out in order to evaluate the antidiabetic properties of *Artemisia afra* Jacq. ex Willd. based on indigenous knowledge which showed that concoctions of the plant can alleviate diabetes mellitus and its complications (Afolayan and Sunmonu, 2011; Dabe and Kefale, 2017; Lutgen et al., 2020).

The hypoglycemic activity and toxicity effect of aqueous leaf extract of Artemisia afra Jacq. ex Willd. was evaluated in streptozotocin-induced diabetic rats (Sunmonu and Afolayan, 2013). Administration of the leaf extract to the streptozotocin-induced diabetic rats significantly increased their body weight, decreased blood glucose levels, increased glucose tolerance, and improved imbalance in lipid metabolism compared to the control group (Sunmonu and Afolayan, 2013). In addition, it was shown that a 200 mg/kg body weight of the extract produced the optimal hypoglycemic action when compared to a standard drug, glibenclamide, demonstrating the potential of *Artemisia afra* Jacq. ex Willd. to act as an antidiabetic medicinal plant (Sunmonu and Afolayan, 2013). Aqueous extracts of *Artemisia afra* Jacq. ex Willd. increased the levels of glutathione reductase, glutathione peroxidase, superoxide dismutase and glutathione in the liver and kidneys of diabetic rats to normal levels and reduced the levels of lipid-peroxidation products (Afolayan and Sunmonu, 2013). Another study by (Afolayan and Sunmonu, 2011) showed that administration of aqueous extracts of *Artemisia afra* Jacq. ex Willd. significantly reduced glucose levels and increased insulin levels in diabetic rats. The aqueous plant extracts also led to regeneration of pancreatic β-cells as indicated by the restoration of the pancreas/body weight ratio (from 0.13 to 0.23%) to near the normal value of 0.27%. *Artemisia afra* Jacq. ex Willd. extracts also exhibited antioxidant activity as seen by the reduction in the levels of lipid peroxidation products such as malondialdehyde (MDA) (Afolayan and Sunmonu, 2011). The enzymatic activities of glutathione peroxidase, glutathione reductase and superoxide dismutase were significantly improved after treatment with the aqueous extract (Afolayan and Sunmonu, 2011). These findings suggest that *Artemisia afra* Jacq. ex Willd. may play a protective role on tissues by reducing oxidative stress.

A case study in the province of Maniema, Democratic Republic of Congo (DRC) showed that *Artemisia afra* Jacq. ex Willd. and *Artemisia annua* L. herbal tea can treat diabetes mellitus (Lutgen et al., 2020). In this study, it was observed during initial trials that several patients were suffering from diabetes mellitus with fasting blood sugar levels ranging from 180 to 280 mg/ml. Upon treatment with *Artemisia annua* and *Artemisia afra* Jacq. ex Willd. aqueous infusions, their blood sugar decreased significantly to 100–130 mg/ml and were comparable to those of the controls. (Issa and Bule, 2015) orally administered aqueous and methanolic extracts of *Artemisia afra* Jacq. ex Willd. collected from Goba town, southeast of Ababa Addis to alloxan induced diabetic mice and evaluated their antidiabetic effect. Their results showed that blood glucose level was significantly decreased by up to 57% in groups that received doses of 750 mg/kg of aqueous extracts of *Artemisia afra* Jacq. ex Willd. relative to untreated group. In addition, the methanolic extracts of *Artemisia afra* Jacq. ex Willd. significantly lowered blood glucose by 49.8% compared to the control group. Overall, the research articles presented here demonstrate that *Artemisia afra* Jacq. ex Willd. contain important bioactive phytochemicals with antidiabetic properties and can be used in traditional medicine systems to treat type 2 diabetes mellitus.

### *Moringa oleifera* lam.

*Moringa oleifera* Lam. of the *Moringaceae* family is native to northern India, but widely used in many regions of the world including Zimbabwe to treat metabolic diseases because of the presence of bioactive phytochemical compounds and minerals in its leaves, barks and roots (Gopalakrishnan et al., 2016; Hassan et al., 2021). In particular, the leaves are rich in minerals (calcium, potassium, zinc, magnesium, iron and copper), vitamins (including vitamins A, B, C, D, and E) and other secondary plant metabolites such as tannins, sterols, terpenoids, flavonoids, saponins, anthraquinones and alkaloids (Hassan et al., 2021). The leaves are also rich in amino acids, including aspartic acid, glutamic acid, serine, glycine, threonine, α-alanine, valine, leucine, isoleucine, histidine, lysine, cysteine, methionine, arginine, and tryptophan (Gopalakrishnan et al., 2016). Other parts of the plant including barks and roots contain important bioactive phytochemicals with antioxidant, anticancer, anti-inflammatory, antidiabetic and antimicrobial properties. *Moringa oleifera* Lam. seeds have anticoagulant properties and are also extensively used in water treatment (Shan et al., 2017). Studies have shown that *Moringa oleifera* Lam. has antidiabetic properties and can help cure type 2 diabetes mellitus (Nova et al., 2020; Watanabe et al., 2021). The *in vitro* and docking studies conducted recently suggest that the natural polyphenols and flavonoids in *Moringa oleifera* Lam. ethanolic leaf extracts have antioxidant and antidiabetic properties (Chigurupati et al., 2022).

Human and animal studies have shown evidence to support *Moringa oleifera* Lam.‘s antidiabetic properties and have been extensively reviewed (Vargas-Sánchez et al., 2019; Nova et al., 2020). (Kar et al., 2003) evaluated the hypoglycemic activities of the organic parts of *Moringa oleifera* Lam. using alloxan-diabetic albino rats. Application of a single dose of *Moringa oleifera* Lam. stem bark ethanolic extract on alloxan induced diabetic rats resulted in significant lowering of blood glucose and urine sugar to undetectable levels. Bioassay-directed isolation and purification of the methanolic extracts of *Moringa oleifera* Lam. identified N-benzyl thiocarbamates, N-benzyl carbamates, benzyl nitriles and a benzyl ester which were all shown to significantly trigger the release of insulin in rodent pancreatic β-cells, and have cyclooxygenase enzyme and lipid peroxidation inhibitory effects (Francis et al., 2004). The leaves of *Moringa oleifera* Lam. exerted hypoglycemic and anti-hyperglycemic effects probably because of the presence of terpenoids, which appeared to be linked with the stimulation of β-cells and subsequent secretion of insulin (Tende et al., 2011). The antihyperglycemic activity of *Moringa oleifera* Lam. tea was investigated on Wistar albino rats and humans by (Fombang and Saa, 2016). *Moringa oleifera* Lam. tea suppressed glucose elevation in both rats and humans, and lower doses were more effective in reducing hyperglycemia possibility due to inhibition of glucose uptake in the intestinal walls by the phytochemical compounds in the tea extract.

A pilot clinical study on thirty-five diabetic and non-diabetic healthy volunteers in Mali demonstrated that *Moringa oleifera* Lam. powder significantly reduced post-prandial blood glucose in diabetic patients but had no effect on blood glucose of the healthy volunteers (Sissoko et al., 2020). The hypoglycemic effect of *Moringa oleifera* Lam. leaf powder supplemented to the local diet of Saharawi diabetic and healthy subjects was investigated in order to determine its hypoglycemic potential in humans (Leone et al., 2018). Seventeen Saharawi diabetic and ten healthy subjects were randomly administered with a traditional meal supplemented with 20 g of *Moringa oleifera* Lam. leaf powder on two different days. Although the healthy subjects did no show differences in mean glycemic meal response, in diabetic subjects, the mean glycemic meal response with *Moringa oleifera* Lam. leaf powder was lower than with control meal. In addition, the postprandial glucose response of diabetic subjects peaked earlier and at lower concentrations with *Moringa oleifera* Lam. leaf powder than with control meal. Next (Anthanont et al., 2016), investigated the effect of *Moringa oleifera* Lam. powdered capsules on plasma glucose and insulin secretion of ten healthy Thai volunteers, and showed that the powdered capsules had no effect on plasma glucose, but significantly increased insulin section. These results suggest that the powder capsules contain important phytochemical nutrients to treat type 2 diabetes mellitus. Formulating bakery cookies with natural product extracts could be a means to treat chronic diseases because they are popular snacks eaten by many people worldwide. However, cookies contain wheat flour that contributes to hyperglycemic and hyperinsulinimic responses. In this regard (Ahmad et al., 2018), conducted a randomized clinical trial to investigate the effect of incorporating *Stevia rebaudiana* and *Moringa oleifera* Lam. powder in cookies on postprandial glycemia, appetite, palatability, and gastrointestinal well-being in humans. Compared to the control cookies and *Stevia rebaudiana* enriched cookies, the *Moringa oleifera* Lam. enriched cookies significantly improved postprandial glycemia and reduced hunger in subjects (Ahmad et al., 2018). Interestingly, a randomized placebo-controlled study by (Taweerutchana et al., 2017) on thirty-two type 2 diabetic patients fed with *Moringa oleifera* Lam. leaf capsules or placebo for 4 weeks demonstrated that *Moringa oleifera* Lam. leaf capsules had no effect on glycemic control and adverse effects in type 2 diabetes mellitus subjects. Together, these studies showed that *Moringa oleifera* Lam. leaves enriched with local foods could be a hypoglycemic herbal drug for people living with diabetes mellitus and more clinical trials are needed because of conflicting results from different research groups.

Gupta et al. (2012) and Al-Malki and El Rabey (2015) observed a rejuvenation of pancreatic islets after treatment of streptozotocin induced diabetic rats with methanol extracts of *Moringa oleifera* Lam. pods. Further, normalization of markers of oxidative stress which include reduction in the plasma levels of glutathione and increased levels of MDA, and reduced activities of the antioxidant enzymes SOD and catalase were observed in diabetic rats treated with moringa seed powder (Al-Malki and El Rabey, 2015). These observations were attributed to the presence of potent free radical scavengers (glucomoringin, quercetin, kaempferol, ursolic acid, myricetin and chlorogenic acid) that have been shown to reduce oxidative stress in diabetic rats treated with Moringa (Gupta et al., 2012; Ampofo-yeboah et al., 2013; Ali et al., 2015). In addition, there was a reduction in the levels of cytokines (Interleukin-6, Interleukin -1β and tumor necrosis factor α), an indication of normalization of metabolic processes in treated diabetic rats (Al-Malki and El Rabey, 2015). Aqueous extracts of *Moringa oleifera* Lam. inhibited α-amylase and α-glucosidase with IC_50_ values of 52.5 and 33.4 mg/ml, respectively (Khan et al., 2017). The aqueous extracts also resulted in increased glucose uptake by yeast cells, and skeletal muscles in rats, while a decrease in intestinal glucose absorption was observed (Khan et al., 2017). Overall, *Moringa oleifera* Lam. extracts contain bioactive phytochemical compounds that act synergistically to lower blood glucose via different mechanisms making Moringa one of the ideal plant-based solutions to management of diabetic conditions.

### *Aloe vera* (L.) Burm.f.

*Aloe vera* (L.) Burm.f. of the *Asphodelaceae* family is a perennial succulent plant commonly found in many parts of Africa and has been used in traditional medicine for more than 2,000 years because of its various medicinal properties (Surjushe et al., 2008). It exhibits antioxidant, antiulcer, anticancer, anti-inflammatory, anti-atherosclerosis and wound-healing effects (Noor et al., 2017). In addition, *Aloe vera* (L.) Burm.f. lowers blood glucose in diabetic patients, and improves the responsiveness of body tissues towards insulin, thus making insulin more effective (Yongchaiyudha et al., 1996). *Aloe vera* (L.) Burm.f. contain a number of anthraquinones, C-glycosides and resins, its gel has several organic acids and biostimulators with topical healing properties (Iwu, 2014). The plant also contains several flavonoids (aloesin, aloenin and barbaloin), polysaccharides, vitamins, lignins, minerals, glycoproteins, phytosterols, and saponins (Iwu, 2014). As such, *Aloe vera* (L.) Burm.f. has many applications in the health and cosmetic industry. The antidiabetic effect of *Aloe vera* (L.) Burm.f. has been extensively reviewed (Radha and Laxmipriya, 2015; Suksomboon et al., 2016; Haghani et al., 2022). A systematic review of the evaluation of biological properties and clinical effectiveness of *Aloe vera* (L.) Burm.f. suggested that the gel can help people achieve better fasting blood glucose levels, as well as reduce body fat and weight (Radha and Laxmipriya, 2015).

A double-blind randomized clinical trial to evaluate the efficacy of *Aloe vera* (L.) Burm.f. in healing of diabetic foot ulcer (DFU) showed that the gel significantly reduced the ulcer surface compared with the control group which showed no significant difference in terms of the ulcer depth (Najafian et al., 2019). Another double blind randomized controlled trial of seventy-two subjects with pre-diabetes mellitus symptoms showed that *Aloe vera* (L.) Burm.f extracts significantly reduced fasting blood glucose, HbA1C, triglyceride, total cholesterol and LDL-C levels with a concomitant increase in HDL-C level compared to placebo (Alinejad-Mofrad et al., 2015). In an effort to understand the metabolic effect of an *Aloe vera* (L.) Burm.f gel complex on obese individuals with prediabetes or early untreated diabetes mellitus, one-hundred and thirty-six subjects were randomly given the gel complex, while the control group were given a soft capsule composed of natural pigments and excipients (Choi et al., 2013). The authors showed that fasting blood glucose and body weight of the subjects fed with *Aloe vera* (L.) Burm.f. gel complex were significantly lower than those of the control group. A clinical trial of *Aloe vera* (L.) Burm.f. products (UP780 and AC952) in patients with prediabetes/metabolic syndrome showed that the formulations can reverse the abnormalities in fasting glucose and improve glucose tolerance of the subjects (Devaraj et al., 2013). These clinical studies provide significant evidence that show the potential of *Aloe vera* (L.) Burm.f. gel and its products to effectively improve the parameters associated with diabetes mellitus in prediabetes and early non-treated diabetic patients.

(Noor et al., 2017) investigated the role of *Aloe vera* (L.) Burm.f. extract on improvement of insulin secretion and pancreatic β-cell function by morphometric analysis of pancreatic islets in STZ-induced diabetic Wistar rats and showed that oral administration of 300 mg/kg body weight *Aloe vera* (L.) Burm.f. extract to diabetic rats for 3 weeks restored blood glucose levels to normal levels and increased their insulin levels. In addition, morphometric analysis of pancreatic sections showed quantitative gain in number, diameter, volume and area of the pancreatic islets of treated diabetic rats compared to the untreated diabetic rats. This study showed that *Aloe vera* (L.) Burm.f. extract exerts antidiabetic effects by improving insulin secretion and pancreatic β-cell function by restoring pancreatic islet mass in STZ-induced diabetic Wistar rats. (Govindarajan et al., 2021) investigated the antidiabetic effect of *Aloe vera* (L.) Burm.f. carbohydrate fraction on insulin secretion, cell proliferation and inflammation using streptozotocin-induced oxidative stress on RIN-m5F cells of diabetic rats. The *Aloe vera*-treated RIN-m5F cells significantly increased bromodeoxyuridine levels and insulin secretion with a concomitant decrease of tumor necrosis factor α (TNF-α), interleukin 6 (IL-6) and nitric oxide levels. Increase in bromodeoxyuridine levels after *Aloe vera* (L.) Burm.f. treatment is associated with rejuvenation of pancreatic β-cells and this leads to improved insulin production. Additionally, the *Aloe vera* (L.)-treated streptozotocin-induced diabetic rats had lower fasting plasma glucose, glucagon and glucose-6-phosphatase levels. The insulin, hexokinase, and glycogen synthase levels and, glycogen content were improved with doses of the *Aloe vera* (L.) Burm.f. carbohydrate extract. The extracts also inhibited α-amylase and α-glucosidase in a dose-dependent manner with IC_50_ values of 60.44 ± 1.02 and 82.85 ± 1.05 μg/ml, respectively. Together, these results suggest that *Aloe vera* (L.) Burm.f. extract regulates glucose metabolism by activation of glycogenesis and down-regulation of gluconeogenesis. Thus, *Aloe vera* (L.) Burm.f. extracts can be used as an alternative medicine in the alleviation of type 2 diabetes mellitus.

Protein glycation is considered one of the main causes of diabetic complications such as vasculopathy, retinopathy, nephropathy and neuropathy, cataracts and chronic kidney disease because it brings about the formation of advanced glycation end products (AGEs), which modify the structure of proteins and alter enzymatic activity. To prevent the formation of AGEs, (Muñiz-Ramirez et al., 2020), evaluated the inhibitory effect of methanolic *Aloe vera* (L.) Burm.f. extract *in vitro* and showed that the extract can significantly decrease the formation of AGEs, fructosamine, N*ε*-carboxymethyl-Lysine and carbonyl protein. The antiglycation activity of the extract is possibly due to the presence of aloin and aloe-emodin compounds acting synergistically to decrease the formation of AGEs (Froldi et al., 2019). In addition, the methanolic extract significantly reduced the activities of *α*-amylase and *α*-glucosidase and increased the content of thiol groups. These findings suggest that a decrease in formation of AGEs brought about by the *Aloe vera* (L.) Burm.f. plant extract may lead to reduced postprandial glucose and prevent diabetes mellitus complications associated with AGE.

In a clinical trial to determine the potential of *Aloe vera* (L.) Burm.f. in lowering blood glucose, ninety non-insulin dependent diabetic subjects from Punjab Agricultural University and Civil hospitals of Ludhiana, India were subjected to *Aloe vera* (L.) Burm.f. gel powder for 3 months, and further supplemented with gel powder and nutrition counselling for another 3 months (Choudhary et al., 2014). A significant decrease in fasting blood glucose level (by 11.4% and 15.4%) and post prandial glucose level (by 18.5% and 27.8%) was observed in the subjects supplemented with gel powder and counselling after the study. The reduction in blood glucose and lipid profiles of the non-insulin dependent diabetic patients was ascribed to the presence of phytochemicals in *Aloe vera* (L.) Burm.f. powder and nutrition counselling. Another study on effects of *Aloe vera* (L.) Burm.f*.* gel on behavioral functions, oxidative status, and neuronal viability in the hippocampus of streptozotocin (STZ)-induced diabetic rats showed that the hypoglycemic and antioxidative properties of *Aloe vera* (L.) Burm.f. gel are possible mechanisms that improve behavioral deficits and protect hippocampal neurons in diabetic animals (Tabatabaei et al., 2017). In addition, (Deora et al., 2021), showed that *Aloe vera* (L.) Burm.f. extracts are capable of alleviating diabetes mellitus-related complications by lowering lipid profile, and restoration of β-cell function in diabetic rats. In summary, we showed that *Aloe vera* (L.) Burm.f. extracts have antidiabetic effects that are comparable to conventional drugs and can significantly improve the healthcare of patients with type 2 diabetes mellitus.

### *Psidium guajava* L.

*Psidium guajava* L., commonly known as guava, is a native plant of many tropical regions of the world including many southern parts of Africa and South America. Various parts of *Psidium guajava* L. have long been used in folk-lore as a medicinal herb to cure infectious diseases, neoplasm, metabolic diseases, digestive diseases and diabetes mellitus (Chu et al., 2022), and various other ailments such as wounds, cough, ulcers, bronchitis, eyesores and diarrhea (Daswani et al., 2017). *In vitro* and *in vivo* animal studies showed that guava leaf extracts improved blood sugar levels, long-term blood sugar control, and insulin resistance. A recent study by (Chu et al., 2022) showed that the aqueous extracts of *Psidium guajava* L. significantly lowered fasting plasma glucose levels and improved glucose tolerance and insulin sensitivity of diabetic mice. Further, the aqueous extracts of *Psidium guajava* L. increased hepatic glycogen accumulation, glucose uptake and decreased mRNA expression levels of gluconeogenic genes, in addition to increasing the expression of glucose transporter 2 (GLUT2) on the cell membrane of hepatocytes. The extracts also altered the composition of the gut microbiota and increased the enrichment of probiotics. These results indicate that aqueous extracts can alleviate hyperglycemia and insulin resistance of T2DM by regulating metabolism of glucose in the liver and restoring gut microbiota.

(Li et al., 2021) evaluated the *in vivo* hypoglycemic and hepatoprotective effects of dried- and rice-fried *Psidium guajava* L*.* leaf decoctions in diabetic rats and showed a decrease in fasting blood glucose and hemoglobin A1c (HbA1c) of the diabetic rats. Further, upregulation expression of glucokinase (GK), glucose transporter 2 (GLUT2), insulin growth factor-1 (IGF-1), insulin receptor substrate-1 (IRS-1), and insulin receptor substrate-2 (IRS-2) was observed in both dried- and rice-fried *Psidium guajava* L. leaf-treated groups. High-performance liquid chromatography and ultra-performance liquid chromatography-tandem mass spectrometry analysis of the *Psidium guajava* L*.* leaf extracts revealed the presence of a high content of ellagic acid, hyperoside, isoquercitroside, reynoutrin, guaijaverin, and quercetin in the rice-fried extract compared to the dry extract. This finding suggests that the rice-fried extract, unlike the dry, has higher antidiabetic effect because of the processing method. Treatment with *Psidium guajava* L. extract showed a significant reduction in blood glucose and HbA1c levels and a significant increase in plasma insulin levels. (Khan et al., 2013) also showed that treatment of STZ-induced diabetic rats with ethanolic *Psidium guajava* L. leaf extract significantly reduced blood glucose and HbA1c levels and increased plasma insulin levels. Additionally, the activities of carbohydrate metabolizing enzymes were restored by treatment with the extract. The antidiabetic effect of the ethanolic extract of *Psidium guajava* L. leaves is likely due to the presence of flavonoids and other phenolic components present in the extract. Furthermore, (Zhu et al., 2020), showed that *Psidium guajava* L. leaf flavonoids, guaijaverin and avicularin have antidiabetic and liver protective effects in diabetic mice.

In another study, (Beidokhti et al., 2020), investigated the inhibitory activities of *Psidium guajava L.* leaf and bark extracts against yeast α-glucosidase and porcine α-amylase and showed that the leaf and bark extracts significantly inhibited α-glucosidase with IC_50_ values of 1.0 ± 0.3 and 0.50 ± 0.01 μg/ml, respectively and α-amylase with IC_50_ values of 10.6 ± 0.4 μg/ml. The extracts had no effect on glucose-6-phosphatase activity in rat hepatoma H4IIE cells but significantly increased 2-deoxy-D-[1–^3^H]-glucose uptake in C2C12 muscle cells and enhanced triglyceride accumulation in 3T3-L1 cells compared to vehicle (DMSO) and positive control (rosiglitazone). These biological activities can likely contribute to improved glycemic control *in vivo*, indicating that *Psidium guajava* L. may have a beneficial role in the management of type 2 diabetes mellitus.

(Musdja et al., 2017) studied the antidiabetic potential of ethanolic *Psidium guajava* L. extracts on diabetic induced male albino rats and showed that the extracts lowered blood glucose levels of diabetic rats by 28–32% compared to the control. (Luo et al., 2019) isolated polysaccharides from guava leaves and evaluated their antidiabetic effects on diabetic mice induced by streptozotocin combined with high-fat diet. When treated with low-dose or high-dose polysaccharides for 2 weeks, the body weight of diabetic mice was partly recovered compared to the control group. The results indicate that guava leaves could significantly ameliorate body weight loss in diabetic mice. Further, fasting blood glucose of the diabetic mice treated with polysaccharides from guava leaves and acarbose (positive control) were significantly reduced compared with the control. Their findings suggested that polysaccharides from guava leaves could provide health benefits for diabetic patients. (Choi et al., 2021) investigated the effects of guava leaf extract on adipogenesis, glucose uptake, and lipolysis of adipocytes and demonstrated that the extracts inhibits adipogenesis and improves adipocyte function by reducing basal lipolysis and increased insulin-stimulated glucose uptake in adipocytes, indicating the antidiabetic effects of guava leaves. These findings show that *Psidium guajava* L. can lower blood glucose and ameliorate body weight loss in diabetic mice.

Studies have shown that the fruit of *Psidium guajava* L. exert antihyperglycemic and antioxidative effects in streptozotocin (STZ)-induced diabetic rat via activation of key effector molecules of the PI3K/Akt pathway, and phosphorylation of AMPK pathway in liver of diabetic rats (Jayachandran et al., 2018; Vinayagam et al., 2018; Tella et al., 2019). In an effort to understand how *Psidium guajava* L. exerts its antidiabetic and anti-hyperlipidemic effects on STZ-induced diabetic rats (Tella et al., 2019), assessed the activities of glycogen synthase (GS), glycogen phosphorylase (GP) and hormone sensitive lipase enzyme (HSL) using radio-chemical methods. The leaf extract significantly decreased HSL activity in adipose tissue and liver of diabetic rats, and increased glycogen storage and HDL-cholesterol levels. In addition, the leaf extract lowered serum triglycerides, total cholesterol and LDL-cholesterol. In addition (Tella et al., 2022), also showed that the *Psidium guajava* L. extracts ameliorated damage to the pancreatic islets and lowered blood glucose in male Sprague-Dawley diabetic rats due to the presence of phenolic compounds and triterpenes in the extracts. (Shen et al., 2008) investigated the effect of aqueous and ethanol extracts of *Psidium guajava* L. leaves on hypoglycemia and glucose metabolism in type 2 diabetic rats. Diabetic rats were fed with aqueous and ethanol extracts of *Psidium guajava L.* over 6 weeks, and the oral glucose tolerance test (OGTT) and other biochemical properties were conducted after sacrificing the rats. The authors showed that acute and long-term feeding of diabetic rats with *Psidium guajava* L. extracts significantly reduced blood sugar levels compared to the control group. Further, long-term administration of guava leaf extracts also increased the plasma insulin level and glucose utilization in diabetic rats. (El-khalik, 2017) studied the effects of *Psidium guajava* L. leaf extracts and bread fortified with *Psidium guajava* L. leaves, and showed that the fortified bread and leaf extracts significantly attenuated diabetic and acute renal failure symptoms in diabetic rats. Together, these experiments provide evidence to support the antihyperglycemic effect and antioxidant properties of guava extracts and their health function against type 2 diabetes mellitus.

### *Persea americana* mill.

*Persea americana* Mill. or avocado belongs to the flowering plant of the *Lauraceae* family. Avocado fruit, seed and leaves are rich in phytochemicals, vitamins, and micronutrients. Studies on antidiabetic properties of indigenous plants have demonstrated that the leaves and seeds of *Persea americana* Mill. are used in the treatment of diabetes mellitus in many parts of Latin America and Africa (Gondwe et al., 2008; Lima et al., 2012; Kouamé et al., 2019). The antidiabetic activity of *Persea americana* Mill. Is believed to be due to the presence of a number of bioactive phytochemical compounds. Liquid-chromatography electrospray ionization mass spectroscopic (LC-ESI-MS) analysis of ethanolic extracts of avocado fruit and leaves revealed the presence of twenty-six bioactive phytogenic compounds (categorized into fatty acids, sterols, triterpenes, phenolic acids, and flavonoids) (Abd Elkader et al., 2022). (Lima et al., 2012) evaluated the antidiabetic activity of hydroethanolic extracts from *Persea americana* Mill. leaves and the mechanism of action via activation of protein kinase B (PKB/Akt) in streptozotocin-induced diabetic rats. By supplying STZ-diabetic rats with hydroalcoholic extracts of the leaves of *Persea americana* Mill., vehicle and metformin, the blood glucose levels, and metabolic state of the animals were significantly improved. In addition, activation of protein kinase B (PKB) was observed in the liver and skeletal muscle of treated rats when compared with untreated rats. These results indicated that hydroalcoholic extracts of *Persea americana* Mill have antidiabetic properties, and possibly act to regulate glucose uptake in liver and muscles by way of PKB/Akt activation.

In another study, (Ojo et al., 2022), examined the role of *Persea americana* Mill. seeds in attenuating alloxan-induced diabetes mellitus by suppressing oxidative stress, inflammation, and β-cell apoptotic death, and by upregulating glucose uptake by stimulating the PI3K/AKT signaling pathway. Their results showed that administration of aqueous *Persea americana* Mill. seed extracts can promote the activation of the PI3K/AkT pathway and inhibit β-cell death, which may be the primary mechanism by which *Persea americana* Mill. seed extracts promotes insulin sensitivity and regulates glycolipid metabolism (Ojo et al., 2022). A study by (Abd Elkader et al., 2022) showed that both fruit and leaf extracts of *Persea americana* Mill. have high content of polyphenols and exhibited high ⍺-amylase inhibitory activities. The fruit and leaf extracts inhibited ⍺-amylase by 92.13% and 88.95% respectively. These results indicate that the antioxidant properties of *Persea americana* Mill. coupled with its ability to activate the pathways and key enzymes involved in glucose metabolism can help in management and treatment of diabetes mellitus.

### *Lippia javanica* (Burm.f.) spreng.

*Lippia javanica* (Burm.f.) Spreng. of the *Verbenaceae* family has long been used in tropical Africa as indigenous herbal tea, refreshing beverage and food additive based on its perceived health and medicinal properties. (Arika et al., 2015) studied the effect of oral and intraperitoneal administration of aqueous leaf extracts of *Lippia javanica* (Burm.f.) Spreng. on blood glucose levels in alloxan induced diabetic mice and demonstrated that the aqueous leaf extracts significantly lowered blood glucose levels in diabetic mice compared to controls. The *Lippia javanica* (Burm.f.) Spreng. aqueous extracts showed hypoglycemic activity in a dose independent manner. The antidiabetic effect of *Lippia javanica* (Burm.f.) Spreng. in diabetic mice was attributed to the presence of a number of secondary metabolites (particularly flavonoids and saponins) acting synergistically as hypoglycemic agents to lower blood glucose. Despite its widespread use as herbal tea to lower blood glucose, there are limited studies in literature to support its antidiabetic action and future work focused on elucidating the antidiabetic compounds and the mode of action of *Lippia javanica* (Burm.f.) Spreng. can help understand its therapeutic benefits.

### *Parinari curatellifolia* planch. ex benth.

*Parinari curatellifolia* Planch. ex Benth. is an evergreen tropical tree of Africa. In Zimbabwe, it is used for faith healing by some indigenous groups and for traditional ceremonies. The seeds of *Parinari curatellifolia* Planch. ex Benth. are commonly used in folk medicine for the treatment of diabetes mellitus and other diseases. (Ogbonnia et al., 2008) evaluated the biochemical safety and hypoglycemic effects of ethanolic extracts of *Parinari curatellifolia* Planch. ex Benth. seeds in alloxan-induced diabetes mellitus in rats. Their results showed a significant reduction in the plasma glucose and low-density lipoprotein (LDL)-cholesterol levels along with a significant increase in high density lipoprotein (HDL)-cholesterol in the treated diabetic groups compared to the control. These results indicate that *Parinari curatellifolia* Planch. ex Benth. seed concoction can be used as a hypoglycemic agent. In a separate study (Ogbonnia et al., 2011), studied the anti-diabetic effect of a 50:50% mixture of ethanolic extract of *Parinari curatellifolia* Planch. ex Benth. seed and *Anthocleista vogelii* Planch. root extract in alloxan-induced diabetic albino rats, and showed that a combination of the two plant extracts was effective in reducing plasma glucose levels in the diabetic rats compared to control group. In addition, significant reductions in LDL-cholesterol, AST and ALT levels and increased HDL-cholesterol were observed in the treated diabetic groups (Ogbonnia et al., 2011). The pancreatic tissue of diabetic rats treated with the extract mixture also showed marked necrotic changes while that of diabetic untreated animals showed more severe β-cell necrosis. These results indicate that the extract mixtures have good hypoglycemic activity and potentially beneficial effects on cardiovascular risk factors.

In another study, the antidiabetic activities of ethanolic stem-bark extract of *Parinari curatellifolia* Planch. ex Benth. was evaluated in *Drosophila melanogaster* fed with high sucrose diet to induce insulin resistance diabetes mellitus (Omale et al., 2020). The high sucrose diet resulted in decreased body size, decreased locomotor activities and delayed emergence of the larva (L3) in flies, all of which are known symptoms of type 2 diabetes mellitus in flies. When administered to the diabetic flies, the extracts of *Parinari curatellifolia* Planch. ex Benth. and standard drugs significantly reduced their glucose levels. Additionally, there was a substantial decrease in the total thiol content, nitric oxide levels, acetylcholinesterase activity and a significant increase in glutathione-S-transferase and catalase activities of the diabetic treated flies.

(Ramashia et al., 2021) investigated the impact of *Parinari curatellifolia* Planch. ex Benth. peel flour on the nutritional, physical, and antioxidant properties of formulated biscuits. Their studies showed that inclusion of *Parinari curatellifolia* Planch. ex Benth. peel flour to biscuit formulations greatly improved the total phenolic and flavonoid content as well as antioxidant activity of the biscuits. The total energy of the biscuits was lowered by ∼8% when the biscuits were enriched with various amounts of *Parinari curatellifolia* Planch. ex Benth. peel flour. Therefore, the formulations of *Parinari curatellifolia* Planch. ex Benth. peel flour could help in reducing weight for overweight/obese persons or in controlling diabetes mellitus. These studies demonstrate the potential of *Parinari curatellifolia* Planch. ex Benth. peel flour as a food additive in reducing diabetes mellitus. Overall, *Parinari curatellifolia* Planch. ex Benth. peel flour has antidiabetic properties because of the presence of polyphenolic compounds and other phytochemical compounds and can be a useful raw material for manufacturing of functional bakery products such as biscuits.

### *Mangifera indica* L.

*Mangifera indica* L. (mango) is tropical plant native to India and Southeast Asia but also widely grown in tropical regions of Africa and Central America (Lauricella et al., 2017). *Mangifera indica* L. contains the polyphenol, mangiferin, a phytochemical compound with hypoglycemic and antioxidant activity (Imran et al., 2017). Oxidative stress has been implicated in the pathogenesis and progression of diabetes mellitus (Giacco and Brownlee, 2010). *Mangifera indica* L. fruit is rich in bioactive phytochemical compounds with antioxidant properties including mangiferin that potentially exerts potent neuroprotective properties against diabetes mellitus-induced oxidative stress. (Cázares-Camacho et al., 2021) investigated effects of *Mangifera indica* L*.* supplemented diet (peel and pulp) on oxidative stress markers in two brain regions (cerebral cortex and cerebellum) of the STZ-induced diabetic rats and observed a decrease in lipid peroxidation in diabetic rats supplemented with *Mangifera indica* L. diet. In addition, the mango supplemented diet reduced polyphagia and weight loss, and maintained a stable glycemia in diabetic rats. These results indicate that *Mangifera indica L.* may exert neuroprotective properties against diabetes mellitus-induced oxidative stress and can be an alternative to prevent and treat diabetes mellitus. A study by (Gu et al., 2019) showed that norathyriol from *Mangifera indica* L. extract significantly inhibited α-glucosidase with an IC_50_ of 4.22 μg/ml. Further, *in vitro* activity assay showed that mangiferin also inhibited α-glucosidase with IC_50_ of 36.84 μg/ml, comparable to acarbose standard (21.33 μg/ml), whereas the IC_50_ value for the whole *Mangifera indica* L. fruit juice, was 112.8 μg/ml, demonstrating the potential of mangiferin and norathyriol to reduce post-prandial glucose level in diabetic patients (Sekar et al., 2019).

Using leaf aqueous extracts on streptozotocin diabetic rats, (El-Sheikh, 2012), observed a significant reduction in serum glucose (∼37.7%) and leptin levels (∼24.3%) accompanied by significant elevation in insulin and C-peptide levels of 28.1 and 24.0%, respectively. Administration of the leaf extract also ameliorated the diabetic effects on asymmetric dimethylarginine (inhibitor of endothelial nitric oxide synthase), Endothelin-1, and serum nitric oxide values. (Saleem et al., 2019) evaluated the potential of *Mangifera indica* L. leaves on postprandial blood glucose, oral glucose tolerance and body weight of alloxan-induced diabetic mice and showed that treated diabetic mice had a remarkable decrease in postprandial blood glucose level compared to untreated diabetic mice. Further, the plant extracts increased glucose tolerance, and body weight, improved lipid profile and decreased the damage to β-cells. The α-amylase and α-glucosidase inhibitory activities of *Mangifera indica* L. leaves was investigated in Nigeria, by (Ojo et al., 2018). The leaf extracts had a considerably high inhibitory effect on α-glucosidase (IC_50_ = 25.11 ± 0.01 μg ml^−1^) and α-amylase (IC_50_ = 24.04 ± 0.12 μg ml^−1^). Electrospray ionization mass spectroscopy (ESI-MS) and high-performance liquid chromatography (HPLC) chemical evaluation of the leaf extract revealed the presence of mangiferin, chlorogenic acid, myricetin, quercetin, rhamnetin, catechin, epicatechin, iriflophenone 3-C-β-D-glucoside and gallic acid (Saleem et al., 2019; Sarkar et al., 2022). Therefore, the inhibitory activities observed in extracts are likely due to the presence of these bioactive antidiabetic agents, and thus *Mangifera indica* L. leaves can be formulated to generate plant-derived nutraceutical drugs to improve human health.

Studies have shown that whole peel powder of *Mangifera indica* L. can help protect from both type I and type II diabetes mellitus (Gondi et al., 2015; Gondi and Prasada Rao, 2015; Irondi et al., 2016). In this regard, (Gondi et al., 2015), studied the effects of mango peel on diabetic rats and showed that urine sugar, urine volume, fasting blood glucose, total cholesterol and triglycerides were significantly reduced in diabetic rats fed with a diet supplemented with mango peel at 5% and 10% levels in the basal diet. In addition, the antioxidant enzyme activities of diabetic rats treated with mango peel increased and the lipid peroxidation in plasma, kidneys and liver decreased compared to untreated diabetic rats (Gondi et al., 2015). Glomerular filtration rate and microalbuminuria were ameliorated in mango peel-treated diabetic group. The peel extract also inhibited α-amylase and α-glucosidase activities, with IC_50_ values of 4.0 and 3.5 μg/ml respectively (Gondi and Prasada Rao, 2015). Another study showed that *Mangifera indica* L. kernel flour improved fasting blood glucose, hepatic glycogen, glycosylated hemoglobin, lipid profile, plasma electrolytes, hepatic and pancreatic malonaldehyde, and the liver function markers of the diabetic rats compared with the diabetic control rats (Irondi et al., 2016). Together, these results indicate that mango peel powder has the potential to function as a therapeutic food component for treatment of diabetes mellitus and management of its complications.

### *Momordica charantia* L.

*Momordica charantia* L (also known as bitter melon, karela, balsam pear, or bitter gourd) is a member of the *Cucurbitaceae* Juss. Family with a wide array of beneficial bioactive phytochemical compounds. It originated in Africa and is a widely grown and consumed vegetable in Asia, East and Southern Africa, India, and South America. The bioactive phytochemicals present in *Momordica charantia* L. include flavonoids, alkaloids and polyphenols in addition to vitamins and minerals which all contribute to its remarkable versatility in treating a wide range of ailments. The potential of *Momordica charantia* L*.* to modulate blood glucose has received greater attention from researchers studying natural foods or compounds that are useful in the treatment of diabetes mellitus and have extensively been reviewed (Lucas et al., 2010; Joseph and Jini, 2013; Peter et al., 2019; Liu et al., 2021; Oyelere et al., 2022; Çiçek, 2022). Various parts of the plant have been shown to possess hypoglycemic properties in many animal models and cell-based assays, while a limited number of human clinical trials have been conducted.

In a study investigating the antidiabetic activities of *Momordica charantia* L. on streptozotocin-induced type 2 diabetes mellitus in rats, it was shown that the fruit juice induced a significant increase of serum insulin, HDL-cholesterol, total antioxidant capacity levels, β cell function percent, and pancreatic reduced glutathione (GSH) content and improved histopathological changes of the pancreas (Mahmoud et al., 2017). In addition, *Momordica charantia* L. fruit juice increased glucose uptake by diaphragms of normal and diabetic rats in the absence and presence of insulin (Mahmoud et al., 2017). *Momordica charantia* L*.* is thought to exert its anti-glycemic property via direct action on β cells of the pancreas and on the intestinal absorption of dietary glucose and amino acids. In a recent study, normal and hyperglycemic Sprague Dawley rats were fed on skin, flesh and whole fruit of *Momordica charantia* L*.* and assessed for diabetes mellitus prophylaxis and treatment. It was shown that a whole fruit juice resulted in ∼31.6% lowering of blood glucose level and ∼27.4% increase in insulin level in hyperglycemic rats (Mahwish et al., 2021). In another study, (Mahwish et al., 2017), conclusively showed that skin, flesh and whole fruit of *Momordica charantia* L*.* significantly increased insulin production and high-density lipoprotein levels with a subsequent decrease in blood glucose, low density lipoprotein and triglycerides in male Sprague Dawley rats. Accordingly, *Momordica charantia* L. possesses hypoglycemic properties and can be a useful additive in the food industry to alleviate medical conditions resulting from complications in blood glucose metabolism.

A double-blind study to compare the antihyperglycemic potential of GlycaCare-II (a herbal formulation of *Momordica charantia* L*.* and other plants) to metformin drug showed that GlycaCare-II and metformin significantly reduced glycosylated hemoglobin (HbA1c) in prediabetic and newly diagnosed diabetic patients (Majeed et al., 2021). Furthermore, GlycaCare-II showed better results for postprandial blood sugar compared to metformin, while a comparable reduction in fasting blood sugar was observed. These clinical trial results suggested that GlycaCare-II may be effective in managing type 2 diabetes mellitus in prediabetic and diabetic subjects. In clinical trials of twenty-four patients who received *Momordica charantia* L*.* or placebo for 3 months, it was shown that *Momordica charantia* L. administration reduced glycated hemoglobin A1c, blood glucose, body weight, BMI, fat percentage, and waist circumference, with an increment of insulin AUC, first phase and total insulin secretion (Cortez-Navarrete et al., 2018). Further, a double-blinded, placebo-controlled, clinical trial of twenty-four type 2 diabetic subjects supplied with *Momordica charantia* L. juice or placebo for 3 months showed a reduction in body weight, HbA1c and blood serum glucose, and increase in insulin secretion of the experimental subjects compared to placebo (Cortez-Navarrete et al., 2018). In addition, it was shown in another randomized clinical study that the hypoglycemic effect of *Momordica charantia* L. was weaker than that of glibenclamide but ameliorates the diabetes mellitus associated cardiovascular risk factors more favorably than glibenclamide (Rahman et al., 2015). (Boone et al., 2017) investigated the effect of acute ingestion of a beverage containing *Momordica charantia* L*.* on blood glucose regulation during an oral glucose tolerance test of ten prediabetic subjects. Interesting, acute ingestion of bitter melon beverage led to the reduction of postprandial glucose in only 50% of the subjects. However, the beverage did not affect insulin response of all subjects. Next, a randomized placebo-controlled single blinded clinical trial of fifty-two prediabetic subjects in Tanzania fed with *Momordica charantia* L*.* powder led to a decrease in fasting plasma glucose of prediabetic subjects compared to placebo (Krawinkel et al., 2018). A randomized placebo-controlled study to determine the efficacy and safety of *Momordica charantia* L*.* as an adjuvant treatment in ninety-six Korean subjects with type 2 diabetes mellitus conclusively demonstrated that the HbA1c levels of patients treated with *Momordica charantia* L*.* and placebo were unchanged, while the fasting glucose levels were significantly reduced in *Momordica charantia* L*.* treated group (Kim et al., 2020). The extracts had no adverse effects on the patients. These clinical studies indicate that *Momordica charantia* L. contain bioactive phytochemical compounds with antidiabetic properties capable of ameliorating diabetes mellitus-induced complications in humans.

### *Annona stenophylla* engl. & diels

*Annona stenophylla* Engl. & Diels is a low-growing perennial plant with woody rhizomes that spread underground with shoots that can rise up to 1 m tall. It is a member of the Annonaceae, custard apple, or soursop family. *Annona stenophylla* Engl. and Diels has been identified in the southern Africa regions including Angola, Botswana, the Democratic Republic of Congo (DRC), Mozambique, Namibia, Zambia, and Zimbabwe. It has been documented to have wide medicinal uses including treatment of abdominal and muscle pains, anemia, malaria, and a range of sexually transmitted diseases among other ailments (Maroyi, 2019). The roots have been used as antidotes and snake repellents (Maroyi, 2019). (Verengai et al., 2017) studied the effects of ethanolic extracts of *Annona stenophylla* Engl. and Diels on alloxan-induced diabetic rats, and observed a dose dependent decrease of plasma glucose levels of the diabetic rats. The most reduction of plasma glucose was achieved by a combination of *Annona stenophylla* Engl. and Diels*, Zingiber officinale* Roscoe*,* and *Citrus limon* (L.) Osbeck. (Taderera et al., 2019) used muscle cell lines C2Cl2 myocytes to investigate the antidiabetic activity of aqueous root extract of *Annona stenophylla Engl. and Diels* and its mechanism of action. They observed a general dose dependent increase in glucose uptake that was comparable with the positive control (insulin administration). In addition, *Annona stenophylla* Engl. and Diels and insulin resulted in an increase in the transcription of GLUT4 mRNA (an insulin-regulated glucose transporter protein that regulates glucose uptake into fat and muscle cells), when compared to the untreated control (Taderera et al., 2019).

Another study by (Taderera et al., 2016) showed that the root extract of *Annona stenophylla* Engl. and Diels had antidiabetic activity that was comparable with the positive controls (glibenclamide and insulin) with glucose reductions of 45%, 46% and 63% for plant extracts, glibenclamide and insulin respectively. (Taderera et al., 2015) also showed that *Annona stenophylla* Engl. and Diels aqueous root bark extracts inhibited both α-glucosidase and α-amylase enzymes with the inhibition not statistically different from acarbose, the positive control. These results showed that *Annona stenophylla* Engl. and Diels plant extracts had comparable effects on the inhibition of both α-glucosidase and α-amylase enzymes as commercial antidiabetic formulations suggesting their potential as antidiabetic agents.

## Why do we need to use crude plant extracts in Africa?

Many valuable African natural plant products have been used for centuries in traditional medicines for the management and treatment of diseases, improvement of human and animal health and nutrition. Africa’s wealth of biodiversity and knowledge of indigenous plants and their products represents an area that remains largely unstudied and undocumented. Even though 80% of the population in the developing countries is estimated to use traditional medicines, the knowledge is undocumented and only a few individuals in communities understand the health benefits of the natural products. These include the elderly and traditional healers and the knowledge is usually passed from one generation to the next through oral means and usually depending on the closeness and interest in use of traditional medicines. Poverty in the majority of the populations living in rural areas is the major driving factor for using natural products to prevent and treat human and animal diseases.

The increased interest and trade in natural products are becoming more important to rural African communities as part of income generating activities. Therefore, natural products can serve as a driver in economic development, providing communities with sustainable harvesting and judicious use of the natural resources. A crude (unfractionated) plant extract contains a range of structurally diverse and often novel chemical compounds. This is important in that crude plant products from a single plant may potentially treat or prevent a number of diseases. Biological activity is often detected and attributed to a single compound or a set of related compounds produced by the plant. Natural products play a vital role in improving human health and have been drugs of choice despite tough competition from modern medicines due to their safety and efficacy.

Nowadays, there is a growing number of companies that engage in packaging and commercialization of crude plant extracts. These products are relatively cheaper compared to modern drugs and have been extensively marketed in Africa including herbal teas such as zumbani (*Lippia javanica* (Burm.f.) Spreng.) tea in Zimbabwe (Mfengu et al., 2021). COFSOL is a cough medicine made from *Lippia javanica* (Burm.f), produced and marketed in Zimbabwe. Imbiza (a mixture of *Cyrtanthus obliquus* (L.f.) Aiton and *Lippia javanica* (Burm.f.) Spreng.) is a herbal tonic commonly used in South Africa to boost the immune system, and for the treatment of chronic diseases (Mahlangeni et al., 2018). In addition, herbal products such as diabecon have been formulated with natural herbs that help reduce excessive blood sugar and decrease hepatic glucose production and prevent hyperglycemia. Thus, diabecon formulation is relatively cheaper than modern drugs and is very effective in the management of type II diabetes mellitus. Accordingly, making herbal formulations from a combination of crude plant extracts could provide a means to prevent and cure diabetes mellitus in many parts of the world where modern drugs are expensive.

## Future directions

Although a number of techniques are available to characterize phytochemical compounds, their precise identification in crude plant extracts is generally complex because they contain a wide variety of structures. In view of this, many people in developing nations have limited capacity to study medicinal plants before using them for medical purposes. Therefore, conducting research covering the toxicity of crude plant extracts may provide a way to inform people of the health risks associated with use of certain medicinal plants. Thus, before packaging crude extracts and selling them on streets (usually common in many African markets), it is important for governments in developing countries to implement rules and strategies that assess the toxicity of these medicinal plants.

**FIGURE 2:**
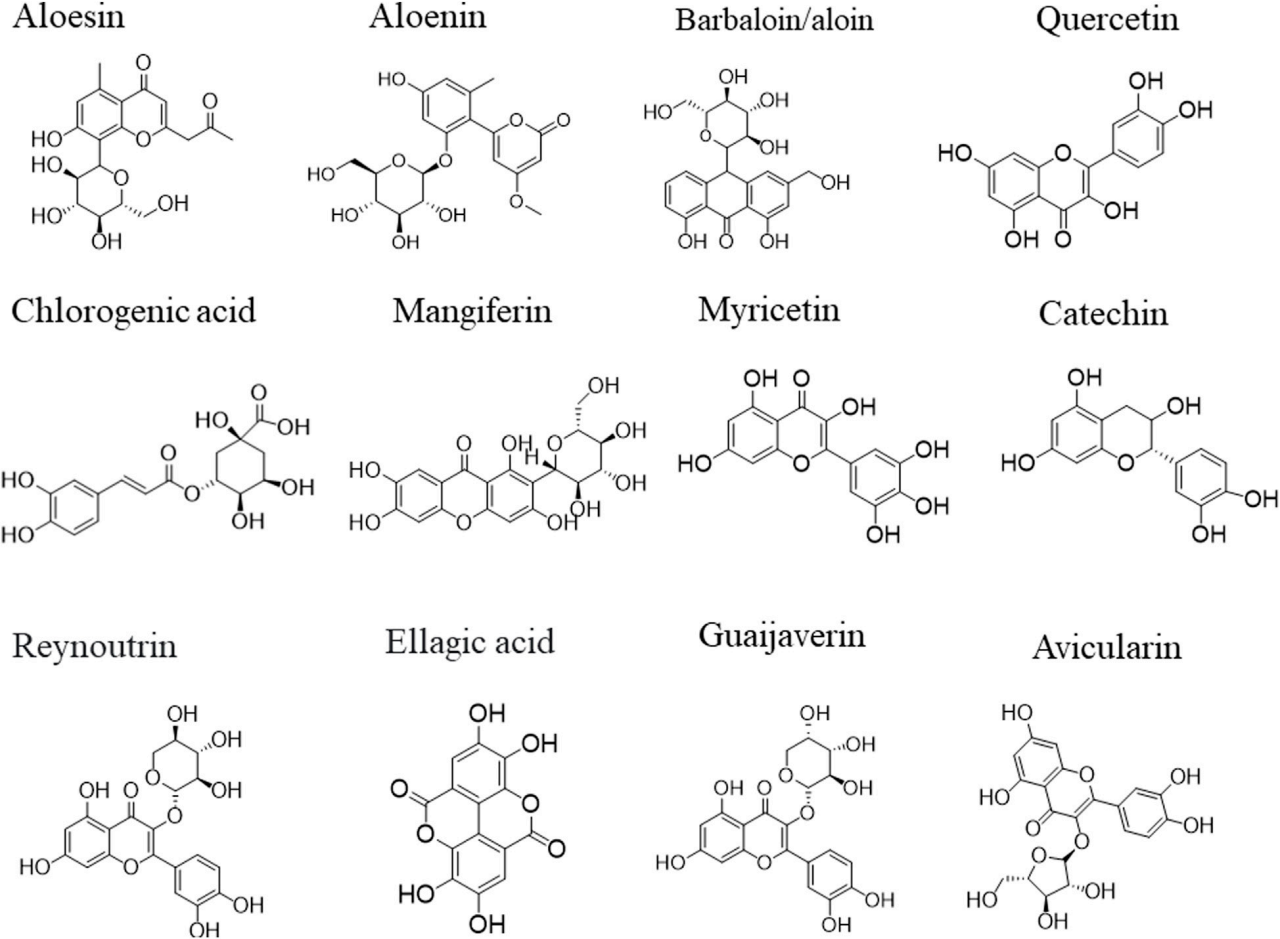
Selected structures of isolated phytochemical compounds with antioxidant and antidiabetic properties found in medicinal plants of Zimbabwe.

A number of phytochemical compounds with antidiabetic properties have been identified using structural tools such as gas chromatography mass spectroscopy (GC MS), liquid chromatography mass spectroscopy (LC MS) or tandem mass spectroscopy (LC MS/MS) and are becoming available in many natural product databases/libraries (Figure 2). In many cases, these compounds have not been fully screened or evaluated for antidiabetic properties. Thus, systematic *in vitro* and *in vivo* studies are needed to evaluate the antidiabetic potential of these compounds guided by computational tools. A variety of powerful computational techniques including protein-ligand docking and virtual drug screening with Autodock tools or Schrödinger Maestro tools have successfully been employed to screen for novel drug leads in many libraries. Thus, future screening of antidiabetic compounds in natural product libraries/databases with computational tools such as Schrödinger Maestro tools and Autodock tools and evaluating their bioactive synergistic characteristics *in vitro* on digestive enzymes (α-amylase or α-glucosidase), GLUT4, GLUT2 and other enzymes involved in regulation of blood glucose can unveil novel compounds for diabetes mellitus treatment. In addition, *in vivo* experiments with novel compounds on humans or animal models can lead to discovery of novel plant-based drug leads for treatment and management of diabetes mellitus. These novel drugs can also be used as prototypes to develop more effective and less toxic drugs for diabetes mellitus treatment by medicinal chemists.

Additionally, there is a growing list of undocumented medicinal plants such as *Xeroderris stuhlmannii* (Taub.) Mendonça & E.P. Sousa that are used frequently to control or treat diabetes mellitus (Selemani et al., 2021). In view of this, it is important for governments to provide funds for research that promotes the use of medicinal herbs to combat many emerging pathogens and diabetes mellitus complications. Finally, even though a number of modern drugs show good hypoglycemic activities, they are often associated with several complications such as nephrological disorders, fatigue, upset stomach and diarrhea. Thus, future work on promoting and regulating research on pharmacological importance of medicinal plants with antihyperglycemic properties and allowing for proper administration of correct doses for crude extracts can be beneficial for ameliorating the various complications associated with diabetes mellitus.

## Conclusion

Awareness about the role of medicinal plants in treatment and prevention of diabetes mellitus will improve health care of many rural populations that rely on herbal remedies for disease control. The use of plants for diabetes mellitus treatment provides an alternative to synthetic drugs as they can be sourced easily and cheaply. Plants contain complex bioactive phytochemical compounds such as polyphenols and flavonoids with many natural bioactive principles and fewer side effects. Thus, determining how these bioactive phytochemicals interact in the human body is important in diabetes mellitus treatment. Therefore, research on this topic may open pathways leading to discovery of new plant derived-drugs, and regularization of use of plant remedies to treat diabetes mellitus by governments across Africa and the world at large.
